# The role of action representations in thematic object relations

**DOI:** 10.3389/fnhum.2014.00140

**Published:** 2014-03-18

**Authors:** Konstantinos Tsagkaridis, Christine E. Watson, Steven A. Jax, Laurel J. Buxbaum

**Affiliations:** ^1^Cognition and Action Laboratory, Moss Rehabilitation Research Institute, Einstein Healthcare NetworkPhiladelphia, PA, USA; ^2^Department of Psychology, School of Humanities and Health Sciences, Neapolis University PafosPafos, Cyprus; ^3^Perceptual-Motor Control Laboratory, Moss Rehabilitation Research Institute, Einstein Healthcare NetworkPhiladelphia, PA, USA

**Keywords:** semantic, action, thematic, taxonomic, apraxia, stroke, object, relations

## Abstract

A number of studies have explored the role of associative/event-based (thematic) and categorical (taxonomic) relations in the organization of object representations. Recent evidence suggests that thematic information may be particularly important in determining relationships between manipulable artifacts. However, although sensorimotor information is on many accounts an important component of manipulable artifact representations, little is known about the role that action may play during the processing of semantic relationships (particularly thematic relationships) between multiple objects. In this study, we assessed healthy and left hemisphere stroke participants to explore three questions relevant to object relationship processing. First, we assessed whether participants tended to favor thematic relations including action (Th+A, e.g., wine bottle—corkscrew), thematic relationships without action (Th-A, e.g., wine bottle—cheese), or taxonomic relationships (Tax, e.g., wine bottle—water bottle) when choosing between them in an association judgment task with manipulable artifacts. Second, we assessed whether the underlying constructs of event relatedness, action relatedness, and categorical relatedness determined the choices that participants made. Third, we assessed the hypothesis that degraded action knowledge and/or damage to temporo-parietal cortex, a region of the brain associated with the representation of action knowledge, would reduce the influence of action on the choice task. Experiment 1 showed that explicit ratings of event, action, and categorical relatedness were differentially predictive of healthy participants' choices, with action relatedness determining choices between Th+A and Th-A associations above and beyond event and categorical ratings. Experiment 2 focused more specifically on these Th+A vs. Th-A choices and demonstrated that participants with left temporo-parietal lesions, a brain region known to be involved in sensorimotor processing, were less likely than controls and tended to be less likely than patients with lesions sparing that region to use action relatedness in determining their choices. These data indicate that action knowledge plays a critical role in processing of thematic relations for manipulable artifacts.

## Introduction

To understand the structure of semantic memory, researchers have worked to uncover the ways in which the concepts of concrete objects can be related to each other. One way in which objects may be related is taxonomically, or within categories of things that share semantic features (e.g., Collins and Loftus, 1975; Rosch and Mervis, 1975; Rogers and McClelland, 2004; O'Connor et al., 2009). For example, taxonomically-related zebras and lions share visual features (e.g., eyes, four legs) and encyclopedic features (e.g., live on the savanna). Another way in which objects may be related is thematically, that is, participating in the same event schema (Nelson, 1983). For example, a golf club and a golf ball are both present in the event of playing golf. While objects related taxonomically have overlapping semantic features (Plaut, 1995; McRae et al., 1997), objects related thematically play complementary roles in a scenario or event (see Estes et al., 2011 for a review). Neuropsychological and neuroimaging research has mapped the distinction between taxonomic and thematic relations to distinct brain areas specialized for processing each type of object relation (Kalénine et al., 2009; Schwartz et al., 2011; Mirman and Graziano, 2012b). For example, Kalénine et al. (2009) found that when participants verified taxonomic relations, bilateral visual areas were activated; by contrast, thematic relations activated bilateral temporo-parietal cortex. Additionally, the production of taxonomic errors during picture naming in aphasia is associated with damage to left anterior temporal lobe while thematic errors in naming are associated with damage to the left temporo-parietal junction (TPJ) and angular gyrus (BA 39) (Schwartz et al., 2011).

Based on this evidence, thematic and taxonomic relations seem to constitute qualitatively different types of semantic information. While taxonomic relations reflect featural overlap between objects (e.g., McRae et al., 1997), there are several ways in which objects can be thematically related. For example, thematic relationships may be based on spatial proximity (e.g., balloon—birthday cake), causal relationships (e.g., fire—ambulance), or common actions between objects (e.g., hammer—nail) (Schwartz et al., 2011; see also Estes et al., 2011). Note that in all of these examples, the two objects participate in a common event; however, the events differ in whether there is direct, physical interaction between the objects. Given such differences, few studies have attempted to uncover which types of relationships are most important for determining thematic similarity between objects (but see Kalénine et al., 2009, 2012).

One clue for understanding the nature of thematic relations comes from recent evidence that taxonomic and thematic relations are differentially important for different kinds of objects (Kalénine and Bonthoux, 2006, 2008). For example, participants are faster to identify taxonomic relations between non-manipulable objects than taxonomic relations between manipulable objects (e.g., poodle—shepherd, sofa—armchair), and faster to identify thematic relations between manipulable objects than thematic relations between non-manipulable objects (e.g., spoon—yogurt, tulip—vase) (Kalénine and Bonthoux, 2008). One explanation of these results is that the privileged status of thematic relations for manipulable artifacts reflects the action knowledge that we have about these objects. Indeed, a growing number of studies suggest that action knowledge is a component of the semantic representations of manipulable artifacts (Helbig et al., 2006, 2010; Myung et al., 2006, 2010; Campanella and Shallice, 2011; Lee et al., 2013). On most accounts of semantic memory, these action features of objects are represented separately from other kinds of semantic features, like color, shape, and typical location (e.g., Allport, 1985; Warrington and McCarthy, 1987; McRae et al., 1997; Barsalou, 1999). Furthermore, the presence of action features for some objects may drive the broad differentiation between manipulable and non-manipulable objects that is observed both in behavioral (e.g., Filliter et al., 2005; Siakaluk et al., 2008) and neuroimaging (see Beauchamp and Martin, 2007 for a review) studies. Thus, for manipulable artifacts, thematic relationships between objects related by virtue of a common action (e.g., hammer/nail) may be more salient than relationships between objects that merely occur within the same event without directly interacting (e.g., hammer and screw co-occur in a “carpentry” event). In the current study, we assessed whether action-based thematic relations are more important for determining the relatedness of manipulable artifacts than non-action thematic relations or taxonomic relations.

If action relationships produce stronger thematic relations, then patients with deficits in action knowledge and/or lesions to regions of the brain involved in the representation of that knowledge (i.e., the left posterior temporal and inferior parietal lobes, e.g., Kalénine et al., 2010) may appreciate action-based thematic relations differently than healthy participants. In general support of this reasoning is a study on the effects of blindness on the organization of object concepts. Connolly et al. (2007) investigated the degree to which congenitally blind participants were implicitly sensitive to information about object color when making similarity judgments between triads of objects. While sighted participants were sensitive to object color when making similarity judgments between fruits and vegetables, blind participants were not. Conversely, neither participant group was sensitive to color information when making similarity judgments about household items. Thus, the inability to access color features prevented color from implicitly influencing blind participants' similarity judgments of fruits and vegetables, a category of objects for which visual semantic features may be especially important. By analogy to the current study, patients with action knowledge deficits may be less sensitive to thematic relations based in action relative to non-action thematic relations or taxonomic relations. Here, we define action knowledge as knowledge of how to physically manipulate objects for their intended uses.

In the present study, we investigated this possibility by comparing object relatedness judgments and the factors which influence these judgments in left hemisphere stroke participants with lesions to posterior temporal and/or inferior parietal cortex, stroke participants whose lesions spare this temporo-parietal region, and healthy participants. The selection of patients with damage in the temporo-parietal region was based on evidence that lesions to inferior parietal and posterior temporal cortex result in deficient object use (apraxia) (e.g., Buxbaum et al., 2007) and recognition of actions (e.g., Kalénine et al., 2010) In addition, neuroimaging studies of manipulable objects and their actions (see Beauchamp and Martin, 2007 for a review) consistently find activation in temporo-parietal areas. In Experiment 1, we developed a novel task to assess the prediction that healthy participants would favor thematic relations based in action over thematic relations not based in action and taxonomic relations between objects. We also used explicit pairwise ratings of action, event, and categorical (taxonomic) similarity to predict participants' judgments of relatedness between objects in a triad. To our knowledge, this is the first comparison of thematic relations with and without action. In Experiment 2, we investigated the prediction that patients with left temporo-parietal damage and/or action recognition deficits (unlike healthy participants, patients with lesions sparing the posterior temporal and parietal region, and/or patients with intact action recognition) would fail to favor thematic relationships based on action in the relatedness judgment task and would not be influenced by pairwise action similarity between objects when making these judgments.

## Experiment 1

Experiment 1 had two aims. The first was to assess the relative strength of healthy participants' preference for thematic relationships entailing an action between objects, thematic relationships not based on action, and taxonomic relationships. The second aim was to model the degree to which the three underlying constructs of event relatedness, action relatedness, and categorical (taxonomic) relatedness determine the choices that participants make. To achieve these aims, we developed 23 object triads in which a reference object was presented with two other objects (termed “active” objects). The nature of the relationship of each active object with the reference object was manipulated so that participants viewed two possible combinations of object relatedness and selected the active object most closely associated to the reference object. For instance, consider a triad with a reference object “wine bottle” and active objects “corkscrew” and “cheese,” for which the task is to choose whether the corkscrew or cheese is “most associated with” the wine bottle. The relationship of the reference object “wine bottle” to “corkscrew” entails a common event that is based on action (opening a wine bottle). The relationship of the reference object “wine bottle” to “cheese,” on the other hand, entails a common event (a wine-and-cheese-party event) but no common action (i.e., cheese is not used upon a wine bottle). The triad stimuli allowed us to assess the conditional likelihood that participants would choose one type of relation over another, as well as assess the degree to which taxonomic, event, and action relatedness determined those choices.

### Methods

#### Participants

Ten neurologically-intact participants (eight women) participated in this experiment (Mean age: 60.3 years, *SD* = 14.8; Mean education: 15.7 years, *SD* = 2.9). Participants were excluded from the study in cases of a history of traumatic brain injury, neurological disease or condition, history of mental illness requiring hospitalization, or drug/alcohol abuse. Participants were all right-handed and scored above 26 on the MMSE (Mini-Mental State Examination; Folstein et al., 1975). Participants were consented in accordance with the guidelines of the Institutional Review Board of Einstein Healthcare Network and were paid for their participation.

#### Stimuli

Stimuli were triads containing a reference object and two active objects. Each triad contained three objects' color photographs displayed on a white background. Supplementary Material Methods provides details of the norming of stimulus pairs used to develop the triads. Each active object in a triad potentially bore one of four types of relations to the reference object: (1) thematic relations involving action (Th+A), (2) thematic relations not involving action (Th-A), (3) taxonomic relations (Tax), or (4) none of these relations (Unr) (see Table S1 in Supplementary Material for examples).

**Figure 1:**
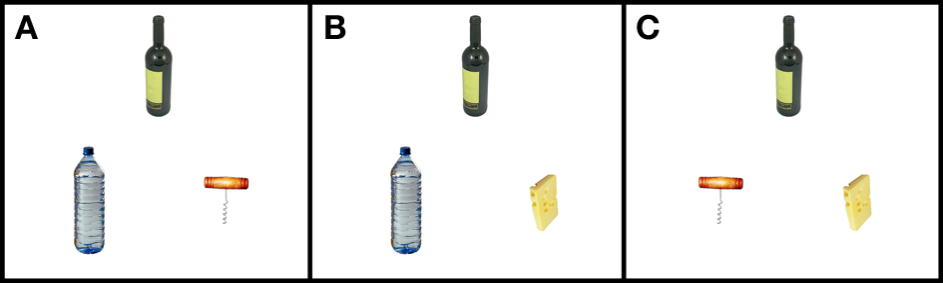
**An example of the three different triad types for one of the reference objects (wine bottle) presented during testing: (A) Th+A vs. Tax (corkscrew, water bottle), **(B)** Th-A vs. Tax (cheese, water bottle), and **(C)** Th-A vs. Th+A (cheese, corkscrew).** The reference object was always positioned at the top and the two possible choices at the bottom of the triad. Each object was coarsely scaled to retain a realistic analogy to the other objects in the triad.

There were three types of triads pitting each type of relation against the other two: Th+A vs. Th-A, Th+A vs. Tax, and Tax vs. Th-A (Figure 1). There were also three triads pitting each type of relation against an unrelated pair: Th+A vs. Unr, Th-A vs. Unr, and Tax vs. Unr. Therefore, there were six triads based on each reference object (23 for each triad type), for a total of 138 trials.

As described in the Supplementary Material, the objects in each triad were normed in a rating study as two sets of pairs, each containing the reference object and another object. Participants in the normative study, who did not participate in the main study, were asked to rate the degree to which the objects in each pair were associated in a common action, event, or category, as well as how visually similar the pair was, and finally, how familiar each individual object was. As described, these ratings were used to select the experimental triads. The Supplementary Material makes clear that the set of triads used in Experiment 1 reflect the types of association that we intended them to reflect: the highest rated set for action association was the Th+A set. The Tax and Th+A sets received the highest category ratings, and the Th+A set received the highest event ratings. Finally, stimuli were matched as closely as possible on familiarity and visual similarity.

### Design and procedure

Triads were presented in random order on a computer monitor. The stimuli were displayed in E-Prime 2.0, using a 21.5″ Acer G215H LCD display. Triads consisted of three objects (300 × 400 pixel resolution images) centered on the corners of an imaginary triangle centered on a 600 × 800 pixel white background image. A single reference object was centered at horizontal midline and within the upper 300 pixels vertically. Two bottom active objects were centered on the ¼ and ¾ points horizontally and within the lower 300 pixels vertically. The position of the two active objects was randomized.

Participants were seated with their eyes approximately 27 inches from the monitor and used the last two buttons on a 5-button box to select the bottom item most closely associated with the reference object (i.e., without specific instructions on how to judge the concept of association). Participants were permitted as much time as necessary to respond.

Prior to the experiment, the experimenter familiarized participants with the individual object images by presenting each on a computer screen along with the written and auditory object name. In addition, participants performed six practice trials (one for each triad type) in which triads were presented and an active object was selected.

### Results

#### Choice data

Overall, active objects having a Th+A relationship to reference objects were chosen most frequently (89%, *SD* = 6.3%), followed by Tax objects (62%, *SD* = 9.7%) and Th-A objects (44%, *SD* = 4.7%). Unrelated objects were rarely selected (5.5%, *SD* = 4.7%) and were thus excluded from subsequent analyses. Additionally, we assessed the context-dependence of participants' choices. In triads requiring a choice between active objects bearing a Th+A or Th-A relationship to the reference object, participants selected the Th+A object an average of 93% of the time (*SD* = 8%). When the choice was between a Th+A and Tax object, the preferred choice was Th+A an average of 76% of the time (*SD* = 13%). When the choice was between Th-A and Tax objects, participants selected the Tax object 65% of the time (*SD* = 18%).

A repeated measures ANOVA revealed that there were significant differences in the percentage of the dominant choice for the three different triad types [*F*_(1.12, 10.10)_ = 8.86, *p* = 0.012; Greenhouse-Geisser correction for violation of sphericity]. Participants made significantly more choices of Th+A pairs over Th-A pairs, as opposed to Th+A pairs chosen over Tax pairs [*t*_(9)_ = 4.8, *p* = 0.001]. They also chose Th+A pairs over Th-A pairs more often than they chose Tax over Th-A pairs [*t*_(9)_ = 3.9, *p* = 0.003]. No other differences were significant. Preliminary analyses including age and gender as covariates in these models indicated that they did not affect the choice data for any of the three triad pairings (all *p*'s > 0.1), and therefore we did not consider these factors in later analyses.

#### Modeling choices based on object pair ratings

To assess the underlying constructs used to inform participants' choices in the triads task, item-level data from each of the three triad types (Th+A vs. Th-A, Th+A vs. Tax, and Tax vs. Th-A) were subjected to three independent hierarchical regression models. The dependent measure for these regressions was a count of the number of participants making the dominant choice for each triad type. For example, in the Th+A vs. Th-A regression, the dependent measure for each triad was the number of participants who chose the Th+A object.

The independent variables at Step 1 were *differences* between the pairwise visual similarity ratings (from the Normative study; see Supplementary Material) of each active object to the reference object, and *differences* between the familiarity ratings (again, from the Normative study) for the active objects. For example, in a triad containing the reference object “wine bottle” and the active objects “corkscrew” and “cheese,” the independent measure for visual similarity, δVisSim, was the Visual Similarity of wine bottle and corkscrew minus the Visual Similarity of wine bottle and cheese. Step 1, then, was a simple model containing ratings on “nuisance” variables known to frequently confound measures of association. Additional independent variables of interest added at Step 2 were differences in the pairwise Category, Event, and Action association of each active object to the reference object. All of these differences were also derived from the ratings data presented in the Supplementary Material. [Note that to clearly distinguish the pairwise ratings data (Supplementary Material) from the stimuli used in Experiments 1 and 2, we use the term “Category” and “Event” when describing the ratings, and “Taxonomic” and “Thematic,” respectively, when discussing the stimuli. The former reflects the “lay” language the participants heard in the normative study when performing the ratings (Supplementary Material), whereas the latter terms make contact with the recent semantic memory literature and are therefore useful from a theoretical perspective (e.g., Estes et al., 2011). Nevertheless, the terms are interchangeable in meaning].

The inclusion of Category, Event, and Action rating differences at Step 2 significantly improved the fit of each of the three models compared to the Step 1 models [Th+A vs. Th-A triads: (Step_1_: *R*^2^ = 0.02, Step_2_: *R*^2^ = 0.41; *R*^2^ change = 0.36, *p* = 0.04); Th+A vs. Tax triads: (Step_1_: *R*^2^ = 0.04, Step_2_: *R*^2^ = 0.41; *R*^2^ change = 0.37, *p* = 0.04); Tax vs. Th-A triads: (Step_1_: *R*^2^ = 0.38, Step_2_: *R*^2^ = 0.84; *R*^2^ change = 0.45, *p* < 0.001)].

Importantly, an examination of each model's coefficients revealed that choices in Th+A vs. Th-A triads were predicted by differences in Action ratings (β = 0.61, *t* = 2.24, *p* = 0.04). For Th+A vs. Tax triads, differences in Category ratings (β = 0.76, *t* = 2.82, *p* = 0.01) predicted choices. Finally, for Tax vs. Th-A triads, differences in Event (β = 0.73, *t* = 4.03, *p* = 0.001) and Visual Similarity ratings (β = 0.53, *t* = 3.93, *p* = 0.001) predicted choices. No other model coefficients reached statistical significance.

To determine whether it was indeed action knowledge that was critical in predicting choices for Th+A vs. Th-A triads, we conducted an additional hierarchical regression analysis with three steps. As before, differences between pairwise Visual Similarity and Familiarity ratings were added at Step 1. Category and Event rating differences were included at Step 2, and Action rating differences were added at Step 3. The improvement of the prediction by the addition of action differences in the 3rd step would verify the critical role of action association in participants' judgments in Th+A vs. Th-A triads, above and beyond any categorical and event-based associations. Indeed, the 3rd step improved the prediction of the choice data as compared to the 2nd step (*R*^2^ change = 0.18, *p* = 0.04).

### Discussion

This experimental paradigm constitutes a novel way of examining the role of action in thematic relations. Analysis of participants' choices in the triads task revealed that objects related by virtue of participation in a common action are deemed strongly associated. In contrast, objects having a thematic relation without direct interaction between them are deemed to be relatively weakly associated, even compared to taxonomic relations, which ranked second in participants' preferences. These data are consistent with prior evidence that thematic associations are processed faster than taxonomic associations among manipulable objects such as tools (Kalénine et al., 2009). However, the present findings further suggest that the privileged status of thematic associations for manipulable artifacts may be conditioned by whether or not the association entails an action relationship.

In further support of the importance of action knowledge in participants' choices, we found that the magnitude of the difference between the action relationship of the reference object and each of the two thematically-related choices in Th+A vs. Th-A triads determined responses. Moreover, in these triads, action relatedness remained a significant predictor of responses even after visual similarity, familiarity, categorical relatedness, and event relatedness were taken into account.

In contrast to the Th+A vs. Th-A triads, the strength of *categorical* associations between reference objects and active objects determined choices between Th+A and Tax objects, and *event-based* association and *visual similarity* determined choices in Th-A vs. Tax pairs. Thus, participants were sensitive to different underlying types of similarity as a function of the relationships that were present in the triads. In other words, the *context* determined the influence of various underlying attributes on participants' judgments of relationship strength. To our knowledge, there is little prior research investigating the role of context in relationship judgments such as those used here. We will return to this point in the General Discussion.

If action plays an important role in the representation of thematic relationships between manipulable artifacts, then influences of action on such relationships should be decreased in patients with deficits in action knowledge and/or damage to the parts of the brain critical for it. We assessed this prediction in Experiment 2.

## Experiment 2

Experiment 2 assessed the performance of patients with lesions to the left posterior temporal and parietal cortex on the triads task (hereafter, the Posterior group) and compared their performance to a patient control group whose lesions spared this region (hereafter, the Anterior group) and to the neurologically-intact control participants included in Experiment 1. Based on the known involvement of the left posterior temporal cortex and inferior parietal lobe in tasks that engage action knowledge (e.g., Kellenbach et al., 2003; Weiss et al., 2008; Kalénine et al., 2010; Randerath et al., 2011), we predicted that the Posterior participants would perform differently than Anterior or Control participants on triads in which Action association is a determining feature, namely, the Th+A vs. Th-A triads. Thus, the Anterior group served as a neurologically-impaired control group, and its members' lesions did *not* include regions predicted to disrupt action knowledge (i.e., inferior parietal and posterior temporal cortex). Additionally, although patients were selected on the basis of their lesions, we also determined whether deficient action knowledge (as assessed by gesture recognition) similarly impacted performance.

If the reported difficulties of temporo-parietal patients with thematic associations (e.g., Mirman and Graziano, 2012b) are based on general difficulty in accessing event-based associations (broadly defined in terms of contextual co-occurrence, independent of action relatedness), we would expect their choices to also differ from the other groups in triads which are primarily differentiated by event ratings (Tax vs. Th-A). If, on the other hand, any observed difficulty with thematic relationships instead reflects the action relatedness of many thematic associates, then the Posterior patients should differ from the other groups only in the Th+A vs. Th-A triads.

### Methods

#### Participants

**Figure 2:**
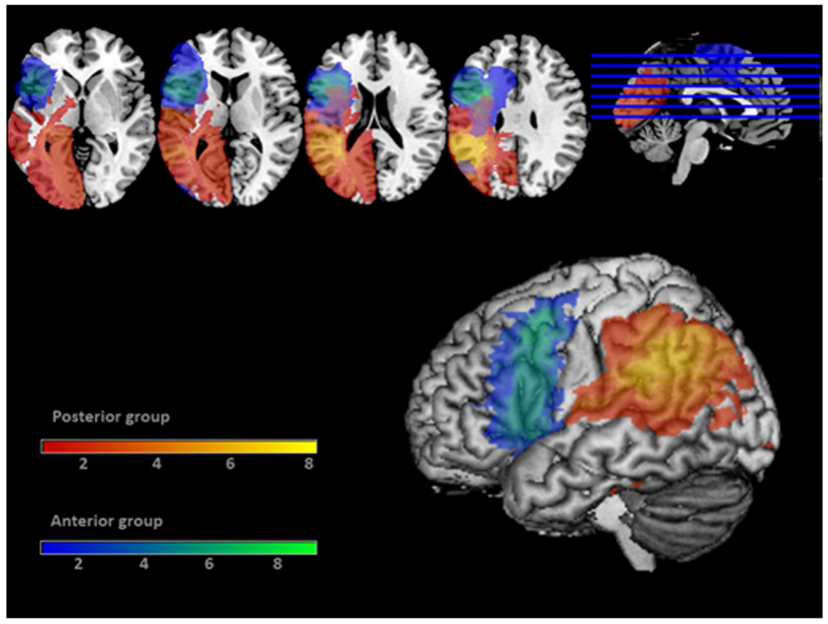
**Coverage map of the lesions of the 17 patients.** Lesions of the Anterior patient group are displayed in blue/green. Lesions of the Posterior patient group are displayed in red/yellow. The 3D rendering displays an 8 mm search depth. Color bars display the number of subjects having lesions at each voxel.

Seventeen left hemisphere stroke participants were recruited from the research registry at Moss Rehabilitation Research Institute. Nine were selected based on the presence of cortical damage to inferior parietal and/or posterior temporal cortex (BA 39, BA 40, superior BA 37, posterior BA 21 and BA 22; Posterior group, see Figure 2). Eight participants were selected who had cortical damage sparing these areas; in this sample, these lesions were largely anterior to the central sulcus (BA 44, BA 45, BA 6, BA 8, BA 9; Anterior group, see Figure 2). All participants were at least 6 months post-stroke and had scores on the Western Aphasia Battery (WAB; Kertesz, 1982) comprehension subtest of at least four points. Additionally, inclusion in the study required participants to be between the ages of 21 and 80 and without pre-morbid or co-morbid neurological brain disease or condition, history of mental illness requiring hospitalization, or alcohol/drug abuse. All participants consented to participate in the study according to the Institutional Review Board of Einstein Healthcare Network and were paid for their participation.

The two patient groups and the neurologically-intact participants from Experiment 1 were roughly equivalent in age [*F*_(3, 47)_ = 2.17, *p* = 0.10] and years of education [*F*_(3, 47)_ = 0.59, *p* = 0.63]. The two patient groups did not differ significantly in lesion volume, though the Posterior groups' lesions tended to be larger [*t*_(15)_ = −1.86, *p* = 0.08]. (There was no evidence for a correlation of total lesion volume and stroke participant choices in the triads task [Tax vs. Th-A: *r*_(15)_ = 0.22, *p* = 0.40; Th+A vs. Tax: *r*_(15)_ = −0.26, *p* = 0.31; Th+A vs. Th-A: *r*_(15)_ = −0.08, *p* = 0.76].) Patients in the Anterior and Posterior groups did not differ in WAB or Gesture Recognition scores. Table 1 displays participant demographics and scores on the Gesture Recognition tasks (see “Action knowledge testing” section).

**Table 1:** **Demographic information about all participants**.

	***N***	***N* females**	**Mean age**	**Mean education**	**Lesion volume (in voxels)**	**Mean WAB score**	**Mean semantic gesture recognition**	**Mean spatial gesture recognition**
Control	10	8	60.3 (14.8)	15.7 (2.9)	–	–	–	–
Anterior	9	5	62.9 (15.8)	15.1 (5.4)	43,547 (24,879)	9.1 (0.5)	91.2 (8.8)	84.2 (9.7)
Posterior	8	3	51.6 (12.7)	15.5 (3.7)	71,433 (29,222)	8.0 (2.1)	87.1 (14.0)	77.8 (17.7)

Standard deviations included in parentheses.

#### Action knowledge testing

Patients were tested with our laboratory's Spatial Gesture Recognition (SpGR) and Semantic Gesture Recognition (SemGR) tasks (Buxbaum et al., 2005; Kalénine et al., 2010). These measures assess the ability to match a spoken and written action name (e.g., “hammering”) to one of two brief videos of an actor pantomiming a skilled tool use movement. Objects are not visible in the videos. In the SpGR task, the incorrect “foil” video differs from the target video by virtue of a spatiotemporal or postural error (e.g., hammering with an open hand). In the SemGR task, the incorrect video is a semantic substitution for the target (e.g., sawing). There were 24 trials each in the SpGR and SemGR tasks. Participants were also given a verb comprehension pre-test to ensure that the action verbs used in the gesture recognition tasks could be matched to an appropriate object photograph (e.g., picture of hammer, written word “hammering”). Any action verbs that were failed on the pre-test were disqualified from the gesture recognition tasks for that participant.

Normative cut-off scores (Spatial Gesture Recognition: 87.1%; Semantic Gesture Recognition: 91.7%; Buxbaum et al., 2005) indicated that for Spatial Gesture Recognition, 56% of Anterior and 62.5% of Posterior patients were abnormal, whereas for Semantic Gesture Recognition 44% of Anterior and 50% of Posterior patients were abnormal. Prior research has found that while gesture recognition impairments are associated with lesions to temporo-parietal cortex, lesions to anterior regions that support general semantic or executive functions may also impair gesture recognition (e.g., middle frontal gyrus, Kalénine et al., 2010). This pattern is evident in the above percentages: although some patients in both groups perform abnormally on Gesture Recognition, a larger percentage of patients in the Posterior group exhibit deficient gesture recognition. Below, we investigate the performance of patients on the triads task based on the location of their lesions and their Gesture Recognition scores; both measures offer a way to examine the intactness of patients' knowledge of actions.

#### Experimental stimuli, design, and procedure

Experimental stimuli, design, and procedure were identical to those used in Experiment 1.

### Results

#### Choice data

As in Experiment 1, we considered the choice data as a function of triad type (Table 2). Data from all participants were analyzed in a mixed-model logistic regression. Because they include both fixed and random effects, mixed models have more power than traditional regression approaches and reduce noise resulting from individual differences. Given prior data showing individual differences in healthy subjects in the selection of thematic and taxonomic choices (Lin and Murphy, 2001; Mirman and Graziano, 2012a), the inclusion of the random effect of participant is particularly relevant to our goal of testing for group differences above and beyond any individual differences.

**Table 2:** **Percentage of participants selecting the dominant choice as a function of triad type**.

**Triad type**	**Choice frequency**		
	**Control (%)**	**Anterior (%)**	**Posterior (%)**
Th+A vs. Th-A	93 (8)	88 (7)	84 (5)
Th+A vs. Tax	76 (13)	78 (8)	80 (9)
Tax vs. Th-A	65 (18)	60 (17)	51 (8)

Standard deviations included in parentheses.

Triad Type (Th+A vs. Th-A, Th+A vs. Tax, Th-A vs. Tax) and Group (Control, Anterior, Posterior) were included as fixed effects, and Participant (*n* = 27) and Item (*n* = 69) were included as random effects. For each participant on each trial, a response was coded in terms of whether it was consistent with the dominant choice for that triad type based on the Control data from Experiment 1. Thus, a choice of a Th+A object always earned a tally of “1” in both of the triad types in which Th+A objects participated (Th+A vs. Th-A and Th+A vs. Tax). In Th-A vs. Tax triads, choices of the Tax object were scored as “1.” These binary choice data were the dependent variable in the logistic regression.

Using this approach, a regression model including the interaction of Triad Type and Group had a significantly better fit than a model including only the main effects [χ^2^_(4)_ = 10.3, *p* = 0.04]. Accordingly, a separate regression was run for each triad type. These analyses revealed a main effect of Group for Th+A vs. Th-A triads [χ^2^_(2)_ = 6.72, *p* = 0.03], but not for Th+A vs. Tax [χ^2^_(2)_ = 0.59, *p* = 0.74] or Tax vs. Th-A triads [χ^2^_(2)_ = 4.32, *p* = 0.11].

The main effect of Group for Th+A vs. Th-A triads was further explored with pairwise comparisons between the groups as implemented in R Software, using the lmer function (generalized linear mixed model fit by the Laplace approximation) of the lme4 package (Bates et al., 2012). Both Controls and Anterior patients were more likely to choose active objects related to the reference object by virtue of a Th+A relationship as compared to the Posterior group, although the comparison between Anterior and Posterior groups was significant only at the level of a trend (Controls vs. Posterior: *z* = −2.37, *p* = 0.02; Anterior vs. Posterior: *z* = −1.67, *p* = 0.09). There was no significant difference between the choices of the Controls and the Anterior group (*z* = −0.70, *p* = 0.49).

Consistent with the pattern seen with the Th+A vs. Th-A triads, ANOVAs conducted separately on the number of each kind of choice regardless of triad type (e.g., the total number of Th+A choices made by each group, overall) revealed that there was a significant group difference for the Th-A choices [*F*_(2, 24)_ = 3.58, *p* = 0.04]. Planned comparisons revealed that participants in the Posterior group made significantly more Th-A choices compared to Controls [*t*_(16)_ = 2.82, *p* = 0.01]. The number of Th-A choices did not differ between the Anterior and Control groups [*t*_(17)_ = 1.04, *p* = 0.32]. As in Experiment 1, addition of age and gender as co-variates in the analyses did not affect the choice data for any of the three triad pairings for any of the participant groups (all *p* > 0.1).

Th+A items were the most frequently chosen by all three groups (Control: Mean = 84%, *SD* = 8.9%; Anterior: Mean = 83%, *SD* = 9.8%; Posterior: 82%, *SD* = 3.3%), followed by Tax (Control: Mean = 44%, *SD* = 14.3%; Anterior: Mean = 41%, *SD* = 11.7%; Posterior: Mean = 35%, *SD* = 7.3%) and Th-A items (Control: Mean = 21%, *SD* = 10.9%; Anterior: Mean = 26%, *SD* = 6.1%; Posterior: Mean = 33%, *SD* = 5.5%). Note that, for the Controls, similar data are reported in the “Choice data” section, but those data included triads with one unrelated object among the alternatives. In the present analysis, we excluded triads with an unrelated object due to the low frequency with which they were chosen; thus, the Control percentages here are slightly different. When expressed as in the “Choice data” section (including triads with unrelated objects) the patient data reflect the following choices: Th+A (Anterior: Mean = 88%, *SD* = 2.6%; Posterior: Mean = 88%, *SD* = 4.2%), Th-A (Anterior: Mean = 47%, *SD* = 8%; Posterior: Mean = 52%, *SD* = 2.8%), Tax (Anterior: Mean = 58%, *SD* = 8%; Posterior: Mean = 54%, *SD* = 5.4%) and Unr (Anterior: Mean = 5%, *SD* = 4.1%; Posterior: Mean = 5%, *SD* = 3.6%).

In short, these analyses revealed that when we considered Th+A vs. Th-A triads, in particular, Posterior patients were less likely to choose the Th+A pair relative to controls. Posterior patients also tended to be less likely than Anterior patients to choose the Th+A option, though this difference was only marginally significant. Finally, Posterior participants made more Th-A choices overall relative to Controls.

#### Modeling choices based on object pair ratings—group differences

Because there was a main effect of Group only for the Th+A vs. Th-A triads, we used the same type of 3-step regression analysis reported in Experiment 1 to determine whether access to action knowledge specifically differentiates the Posterior patients from the other groups on these triads. As in Experiment 1, the 1st step accounted for participant's choices based only on Familiarity and Visual Similarity differences between the two pairs in each triad. The 2nd step included the addition of Category and Event rating differences, and the 3rd step included all of the previous predictors, as well as Action rating differences. This 3rd step again served as the strictest test of the role of action in improving the prediction of participants' choices, above and beyond the influence of other factors. The improvement of fit indicated by the significance of the R square change for each step represents the usefulness of the additional regressor(s) in predicting group choices. We expected that the 3rd step (with the addition of Action rating differences) would improve the prediction of the choices of the Control and Anterior but not Posterior groups.

As shown in Table 3, the 2nd step improved the prediction for the Anterior group's choices, as it had with the Controls in Experiment 1, and, not surprisingly, again for the Control group's choices. Also similar to the controls (albeit less strongly), the prediction for the Anterior group's choices tended toward improvement with the addition of Action association in the 3rd step. In contrast, in the Posterior group, neither the addition of Categorical and Event-based associations (Step 2) nor the full model including Action association (Step 3) was significantly better than the basic model (Step 1) at predicting participants' choices.

**Table 3:** **Hierarchical regression models predicting choices in Th+A vs. Th-A triads**.

**Group**	**Step**	***R*^2^**	***R*^2^ change**	***p***
Control	1	0.02	0.02	0.83
	2	0.20	0.18	0.16
	3	0.38	0.18	0.04
Anterior	1	0.13	0.13	0.26
	2	0.36	0.23	0.06
	3	0.45	0.09	0.11
Posterior	1	0.14	0.14	0.22
	2	0.23	0.10	0.35
	3	0.24	0.002	0.84

The regression coefficients for the full model (Step 3) for each group are presented in Table 4. The critical regression coefficient for Action rating differences (δAct) is a significant predictor of Control choices (β = 0.54, *p* = 0.04), and it is of similar magnitude in predicting the Anterior group's choices as well (β = 0.51, *p* = 0.11). The same coefficient in the Posterior group's model did not reach significance (β = 0.06, *p* = 0.84) and is much lower than the corresponding coefficient for both the Anterior and the Control groups. The magnitude of this coefficient represents a moderate effect size for both the Control and Anterior groups, but it is near zero for the Posterior group.

**Table 4:** **Coefficients of Step 3 model for Th+A vs. Th-A triads; groups defined by lesion loci**.

**Group**	**Control**		**Anterior**		**Posterior**	
	β	***t***	β	***t***	β	***t***
δ Visual similarity	−0.31	−1.86^#^	−0.17	−0.84	0.03	0.16
δ Familiarity	−0.10	−0.67	0.28	1.62	−0.24	−1.37
δ Category	0.33	1.65	0.16	0.64	0.21	0.83
δ Event	−0.28	−1.26	0.13	0.48	0.13	0.47
δ Action	0.54	2.24^*^	0.51	1.70	0.06	0.21

* p < 0.05,

# p < 0.10.

To summarize, choices made by Posterior patients were less predictable in the context of choosing between Th+A and Th-A pairs. Unlike the Controls and Anterior patients, prediction of Posterior patients' choices was not improved by the inclusion of Action rating differences between choices in a triad. This finding is in agreement with the hypothesis of degraded access to action knowledge in patients with temporo-parietal damage.

#### Relationship of Th+A vs. Th-A preferences with action knowledge tasks

To further explore the role of action in thematic associations, we used behavioral tests of the patients' gesture recognition, routinely examined in our lab. With this approach we analyzed the patients' choice data for the Th+A vs. Th-A triads with respect to patients' capacity for gesture recognition rather than lesion location. These tests provide a direct measure of action knowledge, which we assumed to be important for our findings of the differentiation of the Posterior group's choices in Th+A vs. Th-A triads. More specifically, we performed non-parametric correlational analyses assessing the association of Semantic Gesture Recognition (SemGR) or Spatial Gesture Recognition (SpGR) scores, on the one hand, with the percentage of Th+A choices made in the Th+A vs. Th-A triads, on the other hand. The Spearman's correlation of SemGR and Th+A choices trended toward significance (rho = 0.357, one-tailed *p* = 0.079), whereas the correlation of SpGR and Th+A choices was clearly not significant (rho = −0.122, one-tailed *p* = 0.321). Thus, patients with lower SemGR scores (but not patients with lower SpGR scores) tended to make fewer Th+A choices.

We next assessed the prediction that action relatedness would predict Th+A choices in patients with relatively intact action knowledge but not in patients with impaired action knowledge. Using a median split on SemGR scores, patients were divided into high SemGR and low SemGR groups. Two separate 3-step regression analyses of the Th+A vs. Th-A triads were then performed on the data from the high SemGR group and the data from the low SemGR group, similar to the regressions performed on Anterior and Posterior groups. As shown in Table 5, for the high SemGR group, comparisons of Step 2 (containing Category and Event rating differences) and Step 3 (containing Action rating differences) showed a trend toward significant improvement in predicting responses. No such similar trend was evident in the Low SemGR group [High group: *R*^2^ change = 0.14; *F*_(1, 17)_ = 3.26, *p* = 0.09; Low group: *R*^2^ change < 0.001; *F*_(1, 17)_ = 0.002, *p* = 0.97].

**Table 5:** **Coefficients for Step 3 for Th+A vs. Th-A triads; groups defined by semantic gesture recognition scores**.

**Group**	**Low SemGr**		**High SemGr**	
	β	***t***	β	***t***
δ Visual similarity	0.10	0.41	−0.26	−1.03
δ Familiarity	0.13	0.63	−0.08	−0.36
δ Category	0.20	0.78	0.19	0.69
δ Event	0.40	1.44	−0.12	−0.42
δ Action	0.01	0.04	0.53	1.81^#^

# p < 0.10; SemGr, semantic gesture recognition.

### Discussion

Experiment 2 demonstrated that action relatedness was not a significant component of the structure of object similarity relations for participants with lesions to left inferior parietal and posterior temporal cortex. In contrast, action relatedness tended to be used by patients whose lesions spared this region, much as it was by healthy controls. A similar pattern was also observed when patients were divided into groups based on tests of complex action knowledge: patients with deficits in semantic gesture knowledge clearly did not use action in their similarity judgments (*p* = 0.97), much unlike the trend in patients without action deficits (*p* = 0.09), or healthy controls. The data indicate that when action knowledge is weakened or difficult to access, the tendency to appreciate associations between objects having a thematic relationship based in action is accordingly weakened. Although a burgeoning literature is concerned with the role of action in object knowledge (e.g., Kiefer et al., 2007; Bub et al., 2008; Van Elk et al., 2009; Buxbaum and Kalénine, 2010; Peelen and Caramazza, 2012), to our knowledge, this is the first demonstration that the status of action knowledge may influence assessment of the relatedness of multiple objects.

In the General Discussion, we will further explore these and other aspects of the data.

## General discussion

In this study, we have provided several lines of evidence that action plays a prominent role in thematic relations between manipulable artifacts. Data from Experiment 1 showed that objects related by virtue of participation in a common action event are deemed strongly associated, whereas objects having a thematic relation without direct interaction are seen as relatively weakly associated. To our knowledge, this is the first time that these two types of thematic relations have been directly compared to each other. Moreover, within triads of thematically related objects, the differential strength of action relationships between objects contributed to determination of overt responses, even after accounting for visual similarity, familiarity, categorical relatedness, and event relatedness. Finally, the role of action relatedness was specific, evident only when different types of thematic relations were pitted against one another. When, instead, one of the two potential choices in a triad entailed a taxonomic relation, the differential strength of visual similarity and categorical or event-based association determined participants' choices.

Experiment 2 demonstrated that action relatedness did *not* determine thematic similarity relations for participants with lesions to left inferior parietal and posterior temporal brain regions (β = 0.06, *p* = 0.84). In contrast, regression coefficients for the use of action relatedness by patients whose lesions spared this region were moderate in size (β = 0.51), and similar to controls (β = 0.54). A similar pattern was also observed when patients were characterized according to the integrity of action knowledge, this time reaching the level of a trend for the group with relatively spared action knowledge (*p* = 0.09) but far from trend levels for the group with impaired action knowledge (*p* = 0.97). When taken together, the data suggest that when action knowledge is weakened or difficult to access, the tendency to appreciate associations between objects having a thematic relationship based on action may be accordingly weakened. Because differences between Posterior and Anterior groups of patients were only marginally significant, we caution that further research is necessary to validate this interpretation. Yet, the fact that analyzing patients by lesion location *or* Semantic Gesture Recognition scores produced similar results lends support to the claim that action knowledge plays a role in appreciating thematic relations between objects.

Numerous past investigations have focused on the role of action knowledge in object representations. Among the most frequently cited are studies showing motor and premotor activations in functional neuroimaging experiments when pictured objects are viewed (e.g., Chao and Martin, 2000) or named (see Chouinard and Goodale, 2010, for a review). One limitation of such studies, however, is that the observed activations may be epiphenomenal to object recognition. Nevertheless, there are additional indications that action play an important role in semantic object knowledge. Studies of neurologic patients have demonstrated that manipulable artifact naming may be disrupted by tumor loci in the left posterior middle and superior temporal region (Campanella et al., 2010), an area of the brain known to be critically important for action recognition (Kalénine et al., 2010). Additionally, processing is generally more rapid for manipulable artifact pictures when they are preceded by pictures sharing manipulability “features” such as grasp configuration (Helbig et al., 2006). This evidence suggests that action may under some circumstances play a facilitatory role in object processing.

There is also prior evidence that action may play a role in determining competition between objects in an array. When the task is to pick up a specified target object, and target and distractor objects share an action feature (e.g., “compatibility with a power grip”), between-object competition is enhanced, and target selection becomes more difficult (Pavese and Buxbaum, 2002; Botvinick et al., 2009). Recently, eye-tracking studies have demonstrated that visual attention is diverted to distractor objects sharing action features with targets even in the absence of an overt action task. For example, when searching for a named object, participants look longer at distractors sharing a hand posture (e.g., pinch, palm, clench, or poke) with the target than at action-unrelated distractors (Lee et al., 2013). Moreover, participants with deficits in action recognition and skilled object use show a reduction and delay in this competition pattern (Myung et al., 2010; Lee et al., submitted). To this point, however, these effects have concerned action feature overlap; that is, the degree to which two objects evoke the *same* action features. Studies of the processes that may be relevant to relationships between objects used reciprocally in a common action have been remarkably few in number.

Some exceptions to this lack of evidence come from a recent eye-tracking study from our laboratory showing that thematic relationships are processed temporally earlier than relationships based on functional similarity (Kalénine et al., 2012). Thus, for example, when asked to locate target objects such as “broom” in an array, participants look more quickly at thematically-related distractors such as “dustpan” than functionally similar distractors such as “sponge,” even though other types of (non-action) semantic relatedness were equivalent for these two distractor types. As noted earlier, Kalénine et al. (2009) demonstrated that judgments of thematic vs. taxonomic relations for manipulable artifacts, similar to the task reported here, activated the left temporo-parietal cortex. Similarly, De Zubicary et al. (2013) demonstrated left posterior middle temporal and inferior parietal activation in a functional imaging study in which target pictures to be named were paired with thematically-related distractor words to be ignored. In response to such data, we have proposed that thematic artifact relationship processing may frequently entail implicit activation of sensorimotor representations for using the objects together (Kalénine et al., 2012). The present evidence is compatible with these prior results.

The data reported here also have important implications for our understanding of the deficits observed with left posterior temporal-parietal lesions. Lesions in this region are known to be associated with deficits in semantic action knowledge and knowledge of hand/object relationships in the syndrome of apraxia. For example, Kalénine et al. (2010) demonstrated that left temporal-parietal lesions were associated with impaired action recognition in patients with apraxia. Similarly, Buxbaum et al. (2003) showed that left hemisphere-lesioned apraxics had difficulty selecting photographs of hand postures that were appropriate for using pictured objects. Moreover, virtual lesions to the left supramarginal gyrus (SMG) of the inferior parietal lobe disrupted ability to judge the appropriate hand configuration for tool use (Andres et al., 2013), and the SMG is a critical locus of damage in apraxics who position the hand inappropriately for tool use (Randerath et al., 2010). However, the present evidence is the first to suggest that temporal-parietal lesions may affect understanding of object-to-object associations based in action. This represents an extension of prior evidence that left-hemisphere-lesioned apraxics are deficient in semantic knowledge of the manner in which individual objects are manipulated (e.g., Buxbaum and Saffran, 2002). The present evidence is also relevant to claims regarding the “embodiment” or “groundedness” of object processing (e.g., Martin, 2007; Negri et al., 2007; Barsalou, 2008; Gainotti et al., 2013) in suggesting that deficits in the capacity to activate action knowledge in apraxia after left hemisphere stroke have relevance to apraxics' processing of objects. Finally, the present data are an important extension of the work of Connolly et al. (2007) showing that congenitally blind participants fail to implicitly use color information to assess the similarity of fruits and vegetables. Of interest is the fact that, unlike the congenitally blind participants in that study, the Posterior stroke participants in the present study presumably had normal *premorbid* ability to perceive and recognize action. This suggests that the capacity to *currently* represent action knowledge, perhaps in the form of an implicit simulation (c.f., Barsalou, 2008, 2009), may be critical to the observed effects. Future studies assessing the role of action knowledge in the semantic processing of developmental dyspraxics may shed additional light on this possibility.

Prior studies have rarely assessed the distinction between thematic relationships entailing action and other types of thematic relationships (e.g., part-whole relationships or co-occurrence of objects in events). However, it is interesting to note that some (but not all) investigations have suggested that thematic relationships are processed developmentally earlier than taxonomic relationships (Nelson, 1979; Smiley and Brown, 1979; Blewitt and Toppino, 1991; but see Waxman and Namy, 1997). We can speculate that the importance of action knowledge in many thematic relationships may play a role in findings of developmental primacy (see also Kontra et al., 2012). It would be of interest to explore whether there are differences in the developmental trajectory of learning of thematic relationships based in action vs. those, such as part-whole relationships, that may be based in information from vision or other modalities.

An unexpected finding in the present study was the contextually-dependent relevance of different types of association: participants' choices were predicted by different underlying constructs (i.e., Action, Event, Category, and Visual Similarity ratings) depending on the relations present in a triad (Experiment 1). Thus, participants were sensitive to the context in which an object relation occurred. Although few studies have examined the role of context in relationship judgments like those used in the present study, there is evidence from the domain of visual cognition that nearby objects can affect the way in which a target object is perceived. For example, when participants are instructed to detect changes in the color of a target object occurring within an array of distractors, seemingly irrelevant features of distractor objects affect performance (e.g., a distractor changing locations; Jiang et al., 2000). However, effects of nearby objects are not limited to low level visual attributes such as location or color. For example, the likelihood of visually detecting both members of a pair of objects is greater when the objects are typically used together (e.g., key—lock), an effect observed both in neurologically-intact participants (Green and Hummel, 2006) and in patients with neglect and visual extinction (Riddoch et al., 2003, 2010). Finally, healthy participants are more likely to detect members of a pair of taxonomically- or event-related objects (e.g., wheelbarrow—lawnmower) vs. unrelated objects (e.g., wheelbarrow—overhead projector) irrespective of the congruence of the scenes within which these objects are presented (Davenport, 2007).

However, despite extensive research on the role of context in object perception (Biederman, 1972; Pollatsek et al., 1984; Carlson-Radvansky and Irwin, 1995; Chun and Jiang, 1998; Hollingworth and Henderson, 2000; Brockmole and Henderson, 2006; Hommel and Colzato, 2009), we know relatively little about how context affects the computation of different aspects of semantic relatedness. Following prior findings that nearby objects influence the perception of a target object, the present evidence suggests that nearby objects also influence the perceived semantic association of the target to other objects. Further, our findings shed light on a possible mechanism for the faster visual detection of objects that are used together (described above): the presence of an action-based thematic association between objects may prime other semantic features of the objects involved, thus speeding recognition.

We note that a limitation of the current study is the fact that some of the patients in the Anterior control group had lesions to regions that may have disrupted action knowledge. Although Kalénine et al. (2010) primarily found that lesions to temporo-parietal regions impaired gesture recognition, lesions to a small part of the middle frontal gyrus also resulted in impairments. However, lesions to this area also produced impairments on a verb comprehension control task, and the authors conclude that this frontal region may support more general executive processing. In the current study, patients in the Anterior group may have had executive impairments that affected their ability to retrieve action knowledge relevant for judging semantic relations between objects. As a result, the difference between Posterior and Anterior patient groups' choices on Th+A vs. Th-A triads trended toward but did not reach significance. For future research, a better patient control group may be patients with lesions specifically to anterior parts of the temporal lobe. While Schwartz et al. (2011) found that patients with lesions to this area make thematic errors during object naming, anterior temporal cortex is not typically thought to represent knowledge of how to manipulate objects (Ishibashi et al., 2011). Thus, the performance of these patients on thematic relations with and without action may serve as an interesting comparison to patients with posterior temporo-parietal lesions and action knowledge impairments.

In sum, our results demonstrate the importance of action relatedness in thematic relations between manipulable artifacts. Participants favored objects related by a common action over objects that merely co-occurred within the same event, and the strength of this preference was determined by the strength of the action relationships between objects in a triad. Moreover, stroke participants with degraded action knowledge and/or damage to the left temporo-parietal cortex were less influenced by these action relationships relative to control participants and stroke participants without temporo-parietal damage. However, we also found that the specific relations present in a triad affected the kinds of association participants drew upon to make their choices. While action relatedness was highlighted when selecting between thematically-related objects (i.e., action vs. non-action thematic relations), other types of association—categorical (taxonomic) relations and participation in common events—were salient in other triad types. Thus, participants can flexibly engage the different sources of semantic association relevant for a particular context.

## Author contributions

The research questions, hypotheses, and stimulus set were collaboratively developed by Laurel J. Buxbaum and Konstantinos Tsagkaridis. Christine E. Watson, Steven A. Jax, and Laurel J. Buxbaum contributed to data analyses conducted by Konstantinos Tsagkaridis. The paper was drafted by Konstantinos Tsagkaridis, Christine E. Watson, and Laurel J. Buxbaum, with important contributions from Steven A. Jax.

## Conflict of interest statement

The authors declare that the research was conducted in the absence of any commercial or financial relationships that could be construed as a potential conflict of interest.
