# Corrigendum: The role of action representations in thematic object relations

**DOI:** 10.3389/fnhum.2015.00548

**Published:** 2015-09-29

**Authors:** Konstantinos Tsagkaridis, Christine E. Watson, Steven A. Jax, Laurel J. Buxbaum

**Affiliations:** ^1^Department of Psychology, School of Humanities and Health Sciences, Neapolis University PafosPafos, Cyprus; ^2^Cognition and Action Laboratory, Moss Rehabilitation Research Institute, Einstein Healthcare NetworkPhiladelphia, PA, USA; ^3^Perceptual-Motor Control Laboratory, Moss Rehabilitation Research Institute, Einstein Healthcare NetworkPhiladelphia, PA, USA

**Keywords:** semantic, action, thematic, taxonomic, apraxia, stroke, embodied cognition, object relations

We wish to correct a typo on page 7, in the last paragraph of the RESULTS section called “Choice Data” and just before the beginning of the paragraph called “Modeling choices based on object pair ratings-Group differences.”

The mistake is in lines 3 and 5 of this paragraph in the PDF version of the article, where the printed name Th-A should be changed to Th+A. The term Th-A in the last line of the paragraph is correct.

For this correction, the whole paragraph should be written as follows:

“In short, these analyses revealed that when we considered Th+A vs. Th-A triads in particular, Posterior patients were less likely to choose the Th+A pair relative to controls. Posterior patients also tended to be less likely than Anterior patients to choose the Th+A option, though this difference was only marginally significant. Finally, Posterior participants made more Th-A choices overall relative to Controls.”

Original article has been updated.

## Conflict of interest statement

The authors declare that the research was conducted in the absence of any commercial or financial relationships that could be construed as a potential conflict of interest.

