# Supplementary material for: The role of action representations in thematic object relations
Source: Front Hum Neurosci. 2014 Mar 18;8:140. doi: 10.3389/fnhum.2014.00140 (PMC3957493; doi:10.3389/fnhum.2014.00140)
Supplement: Supplementary file 1 [file Presentation1.PDF]

Appendix: Stimulus Norming

A.1. Method

A.1.1 Participants

Twenty-four neurologically-intact participants (17 women, Mean age: 63.8 years, SD = 8.4; Mean education: 16.6 years, SD = 2) consented to participate in accordance with the guidelines of the Institutional Review Board of Einstein Healthcare Network and were paid for their participation.

A.1.2. Stimuli

Stimuli were 38 pairs of objects in which the same reference object was paired with 4 other objects to create 4 types of object relations: a) Thematic relations involving action (Th+A), b) Thematic relations not involving action (Th-A), c) Taxonomic relations (Tax), and d) Unrelated (Unr). More details about the stimuli are included in the Methods section of Experiment 1 (also see Table A-1).

Table A-1: *Stimulus characteristics.*

Relation Type	Active Object	Type of Association	Example
Th+A	▪ Has functional end<br>▪ Used in common action in combination with reference object	▪ Event co-occurrence<br>▪ Physical interaction	Wine bottle – Corkscrew
Th-A	▪ Has functional end<br>▪ Used in same event with reference object, but without direct interaction	▪ Event co-occurrence<br>▪ No physical interaction	Wine bottle – Cheese
Tax	▪ Same taxonomic category as reference object	▪ Semantic category co-occurrence<br>▪ No physical interaction	Wine bottle – Water bottle
Unr	▪ Has functional end<br>▪ Absence of any kind of relation to target object	▪ No semantic category co-occurrence<br>▪ No physical interaction	Wine bottle – Tire iron

A.1.3. Design & Procedure

Participants rated stimuli using explicit instructions and a 1-7 Likert-type scale. Stimulus pairs were displayed within 800 × 600 pixel resolution images (400 × 300 pixels for each object) in E-Prime 2.0, using a 21.5" Acer G215H LCD display. Participants first rated the familiarity of each individual image ("how familiar is the object to you?"). Next, pairs of objects were presented. As indicated in Table A-1, the object pairs consisted of a "reference" object and an additional object that we term the "active" object. For example, one such pair was a wine bottle (reference object) and corkscrew (active object), whereas another such pair was a wine bottle (again, the reference object) and a water bottle (active object). Participants rated each pair for 4 types of association: (1) Categorical ("to what degree do the two objects come from the same category?"), (2) Event-based ("how likely are the two objects to participate in the same event?"), (3) Action ("how likely are the two objects to physically interact?"), and (4) Visual similarity ("how visually similar are the two objects?"). Trials were blocked by rating question, and question order was counterbalanced across participants. Prior to rating, participants were given example trials exemplifying unrelated and highly related pairs. Ratings were not collected for trials in which an image was not recognized (1.8% of trials). Each participant rated approximately half the 152 stimuli (38 reference objects × 4 pairs). Together, two participants rated an entire set of stimuli (see Connolly, Gleitman & Thompson-Schill, 2007 for a similar procedure).

A.2. Results

Based on these data, we selected 23 reference objects and their associated item sets (active objects), matched as closely as possible on Familiarity and Visual Similarity. Action, Event, and Taxonomic ratings differentiated among the sub-sets by design. Only these stimuli were used in Experiment 1.

A.2.1. Familiarity and visual similarity

Equivalence of Familiarity ratings among different object relation types was tested by a one-way ANOVA with Greenhouse-Geisser correction when appropriate. There was a main effect of Object Relation Type [$F(2.7, 54.3) = 4.08$, $p = .013$]. Planned pairwise comparisons revealed that this effect was driven by greater familiarity of the object pairs associated via a Th+A relation, relative to object pairs bearing other types of relations [Th+A vs. Tax: ($t(23) = 2.92$, $p = .008$), Th+A vs. Th-A: ($t(23) = 4.52$, $p < .001$), Th+A vs. Unr: ($t(23) = 3.42$, $p = .002$). Stimuli were also rated for Visual Similarity. There was a main effect of Object Relation Type [$F(2.03, 47.42) = 32.20$, $p < .001$]. Post-hoc analyses showed that this difference was driven, in part, by the greater visual similarity of Taxonomic pairs compared to other pairs [Tax-Th+A : $t(23) = 4.98$, $p < .001$; Tax-Th-A: $t(23) = 11.03$, $p < .001$; Tax-Unr: $t(23) = 8.26$, $p < .001$]. Also, Th+A pairs were more visually similar than Th-A pairs [$t(23) = 3.01$, $p = .006$) and Unr pairs [$t(23) = 2.81$, $p = .01$]. Because Familiarity and Visual Similarity continued to differ between Object Relation types, these factors were used as covariates in the experimental analyses.

A.2.2. Category, Event, and Action similarity

Category, Event, and Action ratings were analyzed in separate repeated measures ANOVAs, with Object Relation Type (4) and Block Order (2) as independent variables.

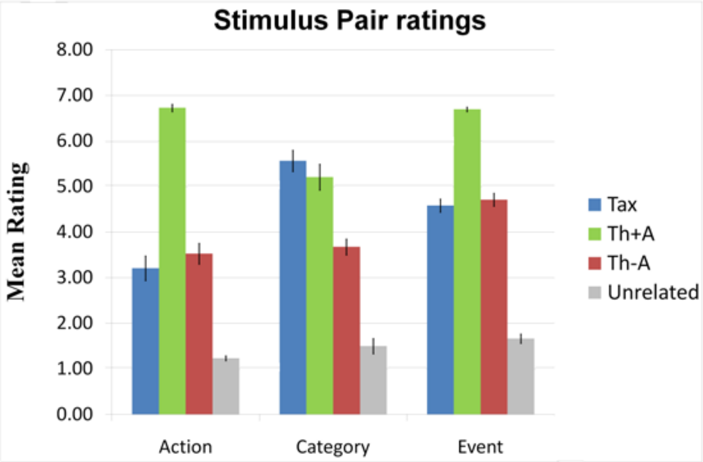

Figure A-1: Mean Action, Category, and Event ratings for each object relation type (Tax, Th+A, Th-A, Unr). Error bars represent +/- 1 standard error.

There was a main effect of Object Relation Type for all rating questions [F_{Cat} (2.24, 44.84) = 113.24, $p < .001$; F_{Ev}(2.16, 43.20) = 352.75, $p < .001$; F_{Act}(2.06, 41.20) = 192.63, $p < .001$]. Planned comparisons revealed significant differences among most of the Object Relation Types on Category, Event and Action ratings (Table A-2).

Table A-2. *Pairwise comparisons of mean ratings of Object Relation Type stimulus sets.*

**Triad Type**	**Category**		**Event**		**Action**	
	d (SD)	*t (23)*	*d (SD)*	*t (23)*	*d (SD)*	*t (23)*
Th+A - Th-A	1.48 (1.20)	6.00***	1.98 (0.71)	13.58***	3.22 (1.04)	15.08***
Tax - Th+A	0.34 (1.63)	1.03	-2.13 (0.73)	-14.18***	-3.55 (1.35)	-12.90***
Tax - Th-A	1.82 (1.02)	8.75***	-0.15 (1.10)	-0.65	-0.34 (1.17)	-1.41
Tax - Unr	3.94 (1.14)	16.99***	2.93 (0.80)	17.88***	1.95 (1.20)	7.95***
Th+A - Unr	3.60 (1.45)	12.17***	5.06 (0.56)	44.40***	5.50 (0.49)	54.98***
Th-A - Unr	2.12 (0.78)	13.38***	3.08 (0.71)	21.24***	2.28 (1.08)	10.37***

Note: *** $p < .001$

Visual Similarity ratings were significantly correlated with Category ratings ($r(67) = .57$, $p < .001$). Action ratings were significantly correlated with Category ($r(67) = .31$, $p = .009$) and Event ($r(67) = .865$, $p < .001$) ratings. Finally, Event and Category ratings were also correlated ($r(67) = .40$, $p = .001$). We took these correlations into account in the analyses reported in Experiments 1 and 2 by requiring that a rating of interest in predicting participants' choice in the triads task must improve the fit of a predictive model that included all of the other ratings (see Experiment 1).
